# Severe Pneumococcal Meningitis With Intracranial Hypertension and Cerebral Venous Sinus Thrombosis in a Patient With HIV and Prior Splenectomy

**DOI:** 10.7759/cureus.111234

**Published:** 2026-06-21

**Authors:** Omar Horani, Sarmed Mansur, Stephen Grider, Hani Saad

**Affiliations:** 1 Hospital Medicine, The University of Toledo Medical Center, Toledo, USA; 2 Emergency Medicine, The University of Toledo Medical Center, Toledo, USA

**Keywords:** bacterial meningitis, cerebral venous sinus thrombosis (cvst), hiv meningitis, intracranial hypertension meningitis, meningitis asplenia, streptococcus pneumoniae meningitis

## Abstract

We report the case of a 45-year-old man with a history of HIV infection (on antiretroviral therapy with a preserved CD4 count) and prior splenectomy, who presented with vomiting, diarrhea, confusion, and worsening headache. He was found to have *Streptococcus pneumoniae* meningitis complicated by intracranial hypertension requiring external ventricular drain placement and extensive cerebral venous sinus thrombosis. This case highlights the risk of severe pneumococcal infection in asplenic and immunocompromised patients and underscores the importance of prompt recognition and management.

## Introduction

Bacterial meningitis caused by *Streptococcus pneumoniae* remains a serious infection with high morbidity and mortality, especially in patients with underlying risk factors such as HIV infection and asplenia. While antiretroviral therapy reduces opportunistic infections, encapsulated bacteria remain a major concern. Intracranial hypertension (elevated ICP) and cerebral venous sinus thrombosis (CVST) are well-recognized and potentially serious complications of bacterial meningitis.

Individuals who have undergone splenectomy experience depletion of this B-cell population, reducing both circulating levels of natural IgM and the capacity to mount T-cell-independent antibody responses to capsular polysaccharides. This creates a dual opsonization defect that explains the predisposition to overwhelming infections with encapsulated organisms.

Cerebral vasomotor reactivity was present in 45% of patients on day 1 and was significantly associated with poor outcome [1]. Patients with pneumococcal meningitis are uniquely prone to several cerebrovascular complications, such as arterial stroke up to 30%, cerebral venous thrombosis up to 9%, and intracranial hemorrhage up to 9% [2].

Elevated ICP may have a rapid course, and death due to herniation is most often seen within the first week. ICP is clinically important because it is strongly associated with mortality, cerebral herniation, and poor neurological outcome. As far as mortality, a systematic review found a clear association between elevated ICP, mortality, and bacterial meningitis, with case fatality rates of 10-37% in high-income countries and up to 54% in low-income countries [2-4]. Approximately half of survivors experience neurological sequelae, including focal deficits, hearing loss, epilepsy, and cognitive impairment [2]. Elevated ICP in bacterial meningitis is multifactorial, arising from three principal mechanisms: cerebral edema (vasogenic, cytotoxic, and interstitial), hydrocephalus from impaired CSF resorption, and alterations in cerebral blood flow [5]. These mechanisms are driven by the intense subarachnoid inflammatory response triggered by bacterial components and the host immune system.

CVST is a recognized complication of bacterial meningitis, particularly pneumococcal meningitis. CVST has been reported in 1-10% of adults. Among patients with pneumococcal meningitis, cerebral venous thrombosis occurs in up to 9% of cases [2]. Headache is the most common symptom of CVST, present in nearly 90% of patients, and may be diffuse and progressive or, occasionally, thunderclap-like. Focal neurological deficits (hemiparesis and aphasia), seizures, and altered consciousness occur depending on the location and extent of thrombosis. The primary mechanism of CVST in bacterial meningitis is septic thrombophlebitis, which leads to endothelial injury along with activation of platelets and the coagulation cascade [2].

This report describes a case of pneumococcal meningitis complicated by ICP and CVST in an HIV-positive, asplenic patient.

## Case presentation

A 45-year-old man presented from home with a chief complaint of vomiting, diarrhea, confusion, and severe headache. His past medical history was notable for HIV infection on Biktarvy (bictegravir, emtricitabine, and tenofovir alafenamide) with a CD4 count of 1164 cells/µL (normal range 500-1500 cells/µL), splenectomy following trauma, cannabis use, and a prior history of smoking.

The patient reported onset of watery, non-bloody diarrhea and recurrent vomiting four days prior to admission, with his last episode occurring the night before presentation. He was unable to tolerate oral intake and experienced chills. Three days before admission, he developed a progressively worsening frontal headache, associated with photophobia and phonophobia. He denied any history of recurrent headaches, chest pain, dyspnea, cough, abdominal pain, dysuria, or visual disturbances.

On initial evaluation, there were no signs of focal neurological deficits, with intact pupillary responses, and a Glasgow Coma Scale score of 15. The patient became severely agitated shortly after arrival in the emergency department and subsequently required sedation and endotracheal intubation.

Investigations

On admission, a CT head prior to lumbar puncture revealed no acute intracranial abnormalities (Figure 1). A lumbar puncture was performed, and CNS analysis revealed a total nucleated cell count of 454 TNC/uL, protein of 262 mg/dL, and glucose of <10 mg/dL, consistent with bacterial meningitis (Table 1). Subsequent CSF and blood Gram stains demonstrated Gram-positive cocci in pairs. Blood and CSF cultures grew *Streptococcus pneumoniae* on hospital day 3.

**Figure 1:**
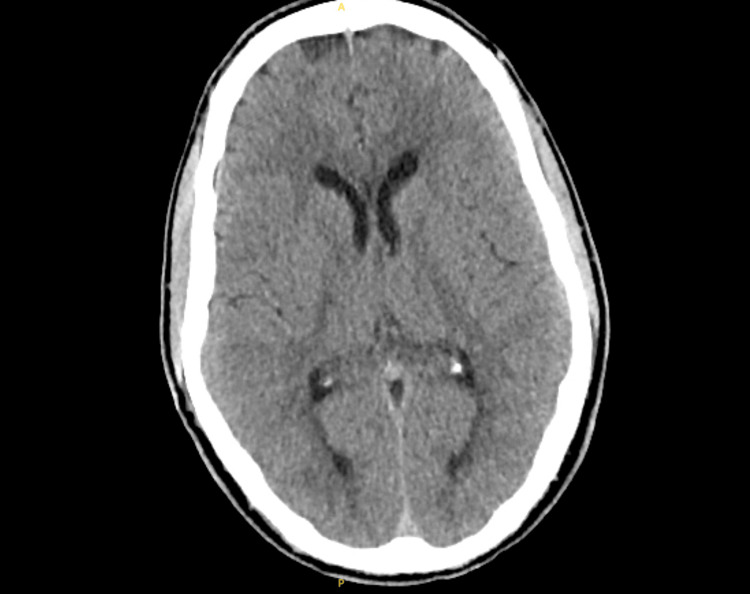
CT head at day 1 showing normal findings with no acute events noted CT: computed tomography

**Table 1:** CSF analysis TNC: total nucleated cells, RBC: red blood cells, CSF: cerebrospinal fluid

	Result	Normal range
Total number of nucleated cells	454 TNC/uL	0-5 TNC/uL
	Neutrophils: 64%	-
	Lymphocytes: 27%	-
	Mono/macrophage: 9%	-
RBC	3495 RBC/uL	0 RBC/uL
Glucose	<10 mg/dL	40-70 mg/dL
Protein	262 mg/dL	20-45 mg/dL
Xanthochromia	Absent	Absent

Management and hospital course

The patient was confused and agitated shortly after arrival in the emergency room, requiring sedation, endotracheal intubation, and admission to the ICU. He was promptly started on intravenous vancomycin, ceftriaxone, and dexamethasone. Despite appropriate antimicrobial and corticosteroid therapy, the patient developed sudden-onset unilateral anisocoria on hospital day 2. Repeat imaging revealed mildly dilated lateral and third ventricles, which were suggestive of elevated ICP (Figure 2). He had received hypertonic saline infusion and mannitol. An external ventricular drain (EVD) was placed by neurosurgery on the same day. An MRI of the brain following EVD placement demonstrated a right frontal ventricular catheter in appropriate position, with no significant ventriculomegaly (Figure 3). Following EVD placement, the initial intracranial pressure, after allowing the waveform to stabilize, was 5-9 mmHg following administration of hypertonic saline and mannitol. On hospital day 3, *Streptococcus pneumoniae* isolated from the blood and CSF culture was susceptible to ceftriaxone. Accordingly, antibiotic therapy was de-escalated to high-dose ceftriaxone. During the ICU stay, the patient showed gradual clinical improvement and was successfully extubated on day 4. Following extubation, the patient reported a severe headache and diplopia with persistent anisocoria. CT venography of the head was done with the suspicion of CVST, which revealed acute dural venous sinus thrombosis involving the superior sagittal sinus and torcula (Figures 4-5). The patient was discharged on intravenous antibiotics and anticoagulation therapy. The patient, who was seen by infectious disease, neurology, and neurosurgery services within three weeks, showed continuous improvement in neurological symptoms.

**Figure 2:**
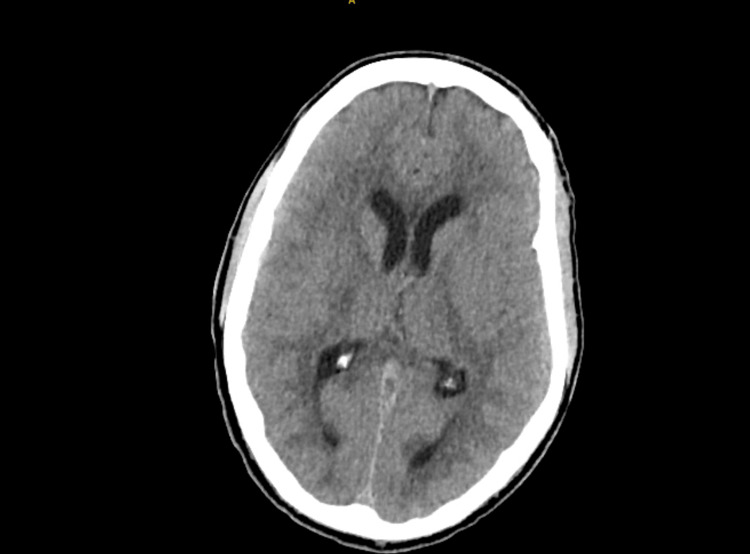
CT head at day 2 showing mildly dilated lateral and third ventricles CT: computed tomography

**Figure 3:**
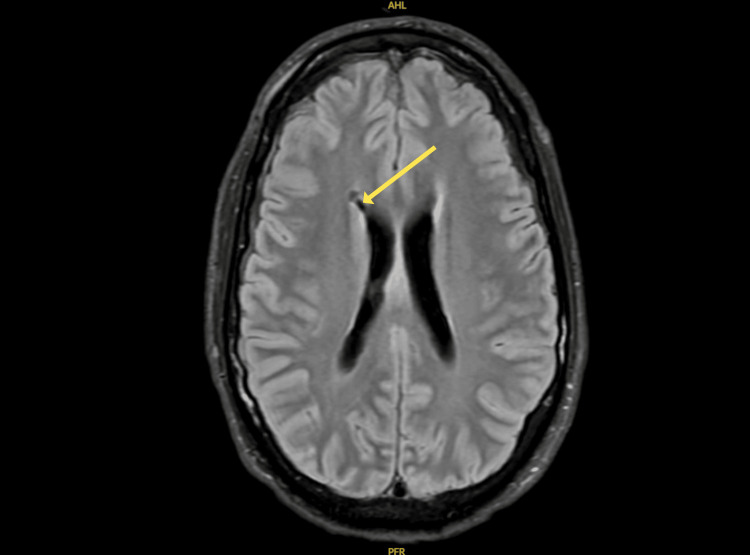
MRI brain at day 3 (T2 FLAIR) showing the tip of EVD (yellow arrow) with no significant ventriculomegaly MRI: magnetic resonance imaging, T2 FLAIR: T2-weighted fluid-attenuated inversion recovery, EVD: external ventricular drain

**Figure 4:**
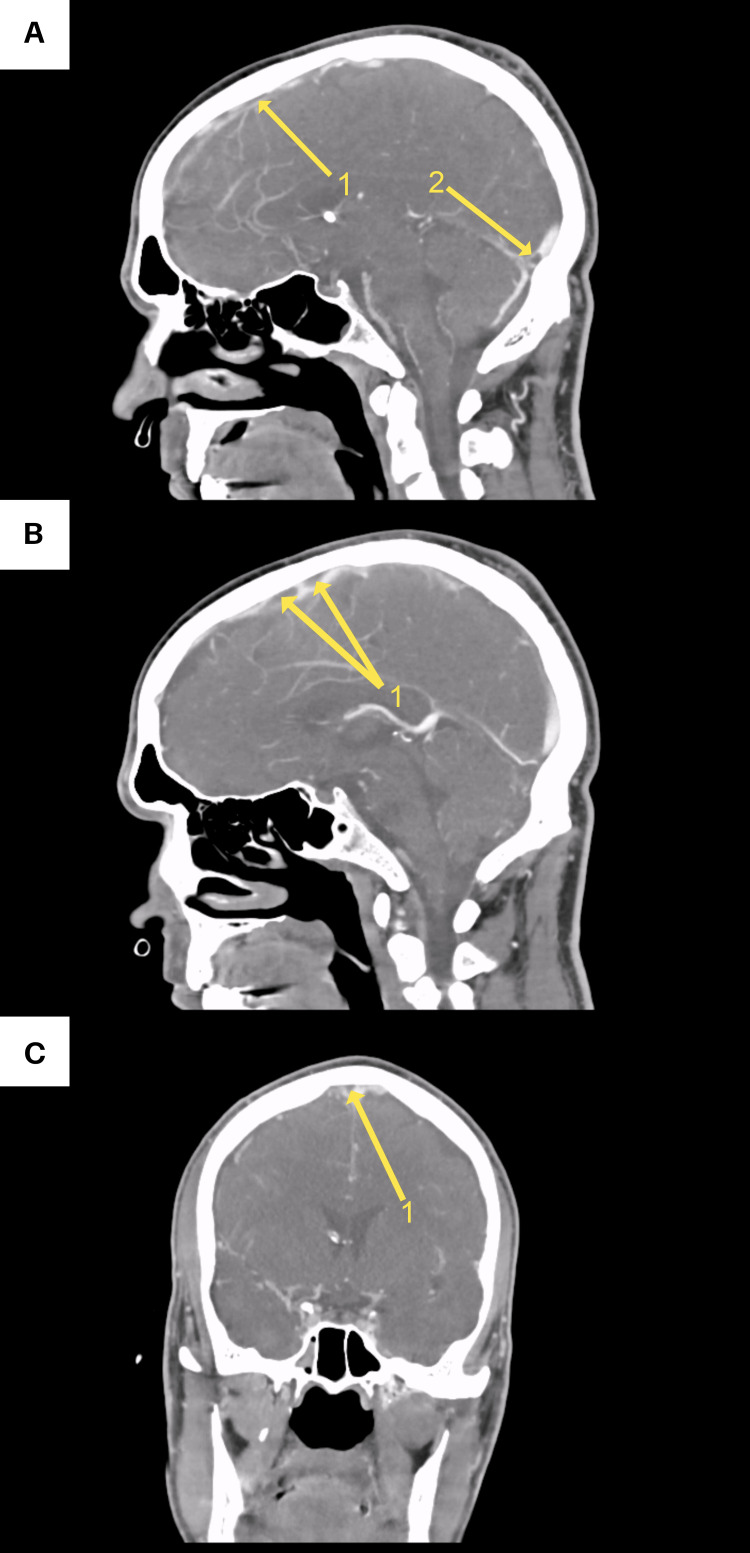
CT venography of the head (A) Sagittal view revealed the superior sagittal sinus (arrow 1) and torcula (arrow 2). (B) Sagittal view revealed superior sagittal sinus thrombosis (arrow 1). (C) Coronal view revealed superior sagittal sinus thrombosis (arrow 1). CT: computed tomography

**Figure 5:**
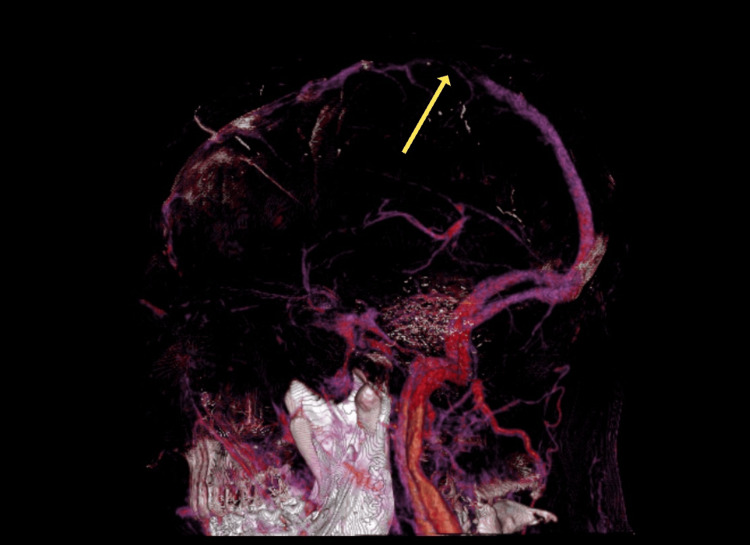
CT venography of the head (sagittal view) revealing the superior sagittal sinus (arrow) CT: computed tomography

## Discussion

This case illustrates the high risk of invasive pneumococcal disease in patients with both HIV infection and asplenia. Although the patient’s CD4 count was preserved, functional immune impairment persists, predisposing to severe bacterial infections. The spleen's marginal zone is uniquely enriched in IgM memory B-cells that produce natural pentameric IgM antibodies [6,7].

The principal opsonins for polysaccharide-encapsulated bacteria, such as *Streptococcus pneumoniae*, whose capsules resist direct complement-mediated recognition, are anticapsular antibodies (both IgG and IgM) and, during the acute phase of infection, C-reactive protein. These opsonins bridge the gap between the capsule surface and phagocyte receptors by binding to the capsule and then activating complement deposition on the capsular surface itself, a critical distinction from complement deposited on the buried cell wall, which is inaccessible to phagocyte receptors.

Splenectomy depletes this B-cell population, reducing both circulating natural IgM and the capacity to mount T-cell-independent antibody responses to capsular polysaccharides, thereby creating a dual opsonization defect that explains the predilection for overwhelming infection with encapsulated organisms [7].

The development of ICP represents a serious and well-documented complication of bacterial meningitis and necessitates timely neurosurgical intervention. A systematic review found that there is a clear association between elevated ICP and mortality, with EVDs being the most commonly used invasive tools for both monitoring and treating elevated ICP [3]. In a prospective cohort of 97 patients with community-acquired bacterial meningitis treated in a neurointensive care unit, 22% had ICP >20 mmHg on day 1, and 46% required CSF drainage; 84% of patients achieved favorable outcomes with this approach [1]. Adult pneumococcal meningitis with ICP requires EVD. The ACIP recommends pneumococcal vaccination for all adults aged ≥50 years (previously 65; lowered in October 2024) and for adults aged 19-49 years with certain underlying medical conditions or risk factors [8]. Ensuring vaccination could significantly reduce the risk of recurrent invasive pneumococcal disease.

Cerebral vascular complications are common in pneumococcal meningitis. Cerebral venous thrombosis occurs in up to 9% and intracerebral hemorrhage occurs in up to 9% [2]. The etiology involves multiple mechanisms, including vasculitis and meningeal inflammation, which cause arterial narrowing and subsequent cerebral ischemia [2,4]. Moreover, persistent pneumococcal cell wall components may serve as a source of ongoing vascular inflammation [9,10]. Brain imaging should be obtained in any meningitis patient with declining consciousness or failure to improve within 48 hours of appropriate therapy.

Anticoagulation in meningitis-associated CVST presents a significant clinical challenge. General CVST guidelines recommend initiating anticoagulation therapy as soon as possible, even in the presence of venous hemorrhage [11,12]. However, empiric initiation of anticoagulant therapy in the absence of CVST should generally be avoided in patients with bacterial meningitis, as it is associated with a high risk of intracerebral hemorrhage [2].

## Conclusions

Pneumococcal meningitis is the most common and severe form of bacterial meningitis in adults, accounting for a large proportion of cases. It is associated with a substantial mortality rate and a significant risk of long-term neurological sequelae among survivors. Individuals with HIV infection and asplenia are at increased risk for invasive infections with encapsulated organisms, particularly *Streptococcus pneumoniae*, which can result in life-threatening pneumococcal meningitis. The European Society of Clinical Microbiology and Infectious Diseases (ESCMID) and the Infectious Diseases Society of America (IDSA) guidelines recommend prompt initiation of empiric therapy with vancomycin plus a third-generation cephalosporin. Ampicillin should be added to cover Listeria monocytogenes in patients >50 years of age or those who are immunocompromised. Adjunctive dexamethasone is recommended in adults with bacterial meningitis, with the benefit most clearly established for pneumococcal meningitis. This case underscores the importance of close follow-up, vaccination, and preventive care in high-risk populations.
